# Effectiveness of multidisciplinary interventions to improve blood culture efficiency and optimize antimicrobial utilization

**DOI:** 10.3389/fpubh.2024.1432433

**Published:** 2024-10-04

**Authors:** Zihuan Li, Keqi Hu, Tian Wang, Baohong Liu, Wen Zheng, Jianqun Zhou, Ting Fan, Maorui Lin, Guanwen Lin, Sujuan Li, Cuiqiong Fan

**Affiliations:** ^1^Department of Infection Prevention and Control, Guangdong Second Provincial General Hospital, Guangzhou, China; ^2^Department of Science and Education, Guangdong Second Provincial General Hospital, Guangzhou, China; ^3^Department of Nursing, Guangdong Second Provincial General Hospital, Guangzhou, China; ^4^Department of Thyroid and Breast Surgery, Guangdong Second Provincial General Hospital, Guangzhou, China; ^5^Department of Laboratory Medicine, Guangdong Second Provincial General Hospital, Guangzhou, China; ^6^Department of Pharmacy, Guangdong Second Provincial General Hospital, Guangzhou, China

**Keywords:** blood culture, multidisciplinary interventions, antimicrobial use, blood culture positivity, blood culture contamination

## Abstract

**Background:**

The low positive rate of blood cultures often leads to downstream consequences. We present a summary of multidisciplinary interventions implemented by a tertiary referral hospital to improve blood culture efficiency and optimize antimicrobial usage.

**Methods:**

We evaluated the knowledge, attitude, and practice (KAP) of healthcare workers in a tertiary care hospital before and after intervention using a questionnaire. A multidisciplinary team was formed to implement the intervention, defining roles, standardizing procedures, continually improving education and feedback, and establishing incentive mechanisms. Regular quality control assessments are conducted on the responsible departments.

**Results:**

Following the intervention, the median submission time for blood culture specimens was reduced from 2.2 h to 1.3 h (*p* < 0.001). Additionally, the intervention group showed significant (*p* < 0.05) increases in rates of positivity (9.9% vs. 8.6%), correct timing (98.7% vs. 89.6%), correct processing (98.1% vs. 92.3%), reduced contamination rates (0.9% vs. 1.4%), and disqualification rates (1.3% vs. 1.7%). The delivery rate of therapeutic antibacterial increased (16.1% vs. 15.2%), and the consumption of restrictive grade antimicrobial also significantly increased (26.7% vs. 22.9%). The intervention measures led to a substantial improvement in awareness and compliance with KAP of blood culture collection in the hospital. Hospital-wide antimicrobial usage deceased by 10.7% after intervention.

**Conclusion:**

A multidisciplinary collaborative model proves effective in improving blood culture efficiency and optimizing antimicrobial usage.

## 1 Introduction

Bloodstream infections are a significant cause of morbidity and mortality worldwide (1, 2). Mortality rates associated with these infections can vary significantly, ranging from 18 to 60% (2). Blood culture is a crucial diagnostic tool for the clinical determination of bacteremia, severe sepsis, and systemic inflammatory response syndrome caused by infections (3). They are essential for confirming infectious etiology, isolating pathogens, determining their drug susceptibility, and guiding targeted therapy (4). Performing blood cultures before initiating antimicrobial therapy in patients presenting with sepsis is critical (5). Blood cultures can identify pathogenic organisms responsible for both community-acquired and hospital-acquired bloodstream infections, allowing for tailored antimicrobial choices for individual patients (6). However, Blood culture contamination is linked to an increased use of antibiotics (7). The overuse of antimicrobials poses risks not only to individual patients (8) but also to entire populations (9, 10) by contributing to antimicrobial resistance. Previously effective antimicrobial agents are becoming less effective, leading to increased mortality rates, extended hospitalizations, and rising healthcare costs (11). Therefore, enhancing blood culture efficiency is a key strategy for optimizing antimicrobial utilization and combating the threat of antimicrobial resistance.

Moreover, the reliability of blood culture can be affected by multiple factors throughout the process, including specimen collection, transportation, and laboratory detection. Blood culture contamination can occur due to the transfer of organisms from the patient’s skin, the immediate environment supplies used for sample collection, or transfer, or the hands of healthcare workers performing the procedure (3). Potential causes for false-negative blood cultures include delays in transportation and processing, inadequate blood volume, and other factors. Interventions to minimize blood cultures contamination have not been studied in isolation. Instead, they are often bundled into multidisciplinary performance improvement projects that typically incorporate education and training, specialized kits, sterile gloves, phlebotomy teams, and other measures (3, 12).

Consequently, multi-departmental collaboration has emerged as a method to enhance blood cultures efficiency and optimize antimicrobial utilization. To this end, we had implemented a multidisciplinary collaboration model that involves the medical, nursing, hospital infection management, clinical laboratory, pharmacy, information technology department, and other interdisciplinary teams. This model enabled us to implement a scientific management approach to explore how to improve blood culture efficiency through various quality control indicators, thereby optimizing the use of antibacterial drugs. We also analyzed the knowledge, attitudes, and practices of medical staff regarding blood culture collection before and after the intervention to further assess the effectiveness of this initiative.

## 2 Materials and methods

The study was conducted as a one-year intervention, divided into before intervention (January to June 2023) and after intervention phases (July to December 2023).

### 2.1 Questionnaire

A knowledge, attitude, and practice (KAP) questionnaire survey regarding blood sample collection for culture was administrated to nurses and doctors. The questionnaire consisted of four sections. The first section (5 questions) assessed personal characteristics of the respondents, including sex, age, educational level, title. The subsequent sections focused on knowledge (30 questions), attitude (12 questions), and practice (12 questions) related to blood culture. The knowledge section comprised of 24 multiple-choice questions and 6 true or false judgment questions. Mean correct responses were calculated to assess knowledge. The attitude and practice sections consisted of 12 items each, where respondents used a Likert scale ranging from 1 (strongly disagree) to 5 (strongly agree) to express their attitudes and practices Mean scores with 95% confidence intervals for attitude and practice were graphically presented using bar charts. To ensure a comprehensive understanding of the study’s topic, objectives, and significance, participants received thorough training from either a nurse practitioner or director before doing the survey. This enhanced the reliability and authenticity of the survey data. An online response system was employed to prevent any missing items, and submissions could only be completed if all required fields were filled. The survey was conducted over 1 week, and the backend data was promptly verified and summarized after the survey was completed.

### 2.2 Multidisciplinary collaboration model

In July 2023, we established a multidisciplinary collaboration team across various hospital departments to enhance cooperation within the hospital. This team is led by the Vice President for Operations and consists of two key components: an expert group and a quality control group. The expert group comprises specialists from diverse fields, including the Nursing Department and medical departments (such as the infection management, quality management, and medical divisions), the laboratory medicine department, the pharmacy department, and clinical department. The quality control team consists of members from the operational backbone of relevant management departments, the information department, the distribution Centre, and quality control doctors from the clinical departments involved in the study. Furthermore, the Nursing Department established a blood culture specimen collection nursing standard management team, which includes liaison officers from the hospital infection team of each ward (Supplementary Table S1).

### 2.3 Standardized processes

This task focuses on enhancing the standardized process and management system for collecting and delivering blood culture specimens in hospitals (Supplementary Figure S1). Additionally, there is a need to create a video demonstrating the standardized collection of blood culture samples for adults or children per each ward. This task will refer to the approved guidelines of principles and procedures for blood cultures published by the Clinical and Laboratory Standards Institute (CLSI), while taking into consideration the specific practices followed at our hospital.

### 2.4 Education and feedback

It is essential to provide clear guideline on the job responsibilities, requirements, and significance of the work performed by the members of the Quality Control (QC) team in the project, as well as their contributions to the overall objectives. Our training sessions for clinical department and transport center staff primarily focus on standardizing the collection and transportation of microbiological specimens. The training content covers the correct methods of collecting microbiological specimens, storage conditions, and precautions for specimen’s transportation. The goal is to ensure consistent and standardized operations among the staff in clinical departments and the transport center.

To monitor and enhance the quality of blood culture, the quality control group conducts monthly meetings to analyze quality control indicators and identify any issues from the previous month. Corresponding solutions are proposed, and responsible departments and individuals are supervised to address and rectify any identified problems. This process aims to continuously improve the quality and efficiency of blood culture procedures. Additionally, the expert group holds quarterly working meetings to improve the quality of all quality control indicators. During these meetings, in-depth discussions and research on existing problems take place, with a focus on enhancing collaboration and communication between different departments. The objective is to promote a more efficient and effective working environment across the organization.

### 2.5 Incentive mechanism

The department has conducted an analysis of the project’s progress, shared and promoted key cases, and implemented a performance management system that incorporates blood culture indicators within the assessment scope. Scores are awarded based on the department’s involvement in blood culture activities, the promptness of data submission, and the effectiveness of quality improvement efforts.

### 2.6 Quality control indicators

Eight essential quality control indicators have been selected, with each indicator being implemented and supervised by the respective department. Specific quality control objectives are established for each indicator to ensure that the blood culture process is performed in accordance with established standards and guidelines.

#### 2.6.1 Specimen submission time

The median submission time of blood culture specimens is determined by dividing the total delivery time by the number of specimens collected within the same period. The quality control target for this indicator is set at 2 h or less. To calculate the specimen submission time, the time recorded by the machine in the laboratory medicine department is subtracted from the time of specimen collection in the clinical department. It is important to note that specimens should be stored at room temperature and should never be refrigerated or frozen, as such conditions may compromise the viability of microorganisms present in the specimens (13, 14).

#### 2.6.2 Positive blood culture rate

The positive blood culture rate is determined by dividing the number of positive blood culture sets by the total number of blood culture sets sent for testing and multiplying the result by 100%. The quality control target is to improve over the previous.

#### 2.6.3 Adult blood culture 2–3 sets per episode rate

The rate of adult blood culture 2–3 sets per episode rate is determined by dividing the number of adult blood cultures sent for 2–3 sets per episode by the total number of adult blood cultures sent for blood culture. The quality control target for this indicator is set at 100%.

#### 2.6.4 Blood culture contamination rate

The blood culture contamination rate is determined by dividing the number of contaminated blood cultures by the total number of routine blood cultures accessioned, and then multiplying the result by 100%. Contaminated blood culture is defined as a microorganism isolated from a blood culture that was introduced into the culture during specimen collection or processing and that was not pathogenic for the patient from whom blood was collected.

#### 2.6.5 Disqualification rate of blood culture specimens

The failure rate of blood culture specimens is determined by dividing the number of sets of failed blood culture specimens by the total number of sets of blood culture specimens in the same period, and multiplying the result by 100%. The quality control target for this indicator is set at less than 5%. Disqualified specimens encompass various situations, including those that are incorrectly labelled or lack the patient’s name, those that do not match the required specimen type and test item, those with containers that are damaged or show serious contamination on the container surface, and those collected using non-compliant containers. Additionally, specimens that do not meet the standards for quality assessment or fail to meet the requirements for collection site, transfer containers, and transfer conditions are also considered disqualified.

#### 2.6.6 Correctness of blood culture collection methods

The videos submitted by clinical department will be evaluated by a team of experts to assess the correctness of blood culture collection methods. The quality control target for this indicator is set at 100%.

#### 2.6.7 Correct timing of collection

Blood cultures should be collected at the onset of fever or chills, or prior to administering antimicrobials (15). The quality control target for this indicator is set at 100%. A monthly sample, equivalent to one-tenth of the medical records with prescribed blood cultures from the previous month, was examined. If there were fewer than 10 cases, all of them were included in the analysis.

#### 2.6.8 Correct handling of positive blood culture results

The percentage of positive blood culture results correctly handled by clinical departments is determined by dividing the number of correctly handled cases by the total number of cases, and then multiplying the result by 100%. Correct handling of positive blood culture results is defined as follows: firstly, the doctor’s assessment of the blood culture results and medication is accurate in the context of the patient’s condition; secondly, when a healthcare professional receives a report of a critical blood culture value, it must be recorded within 6 h; thirdly, the physician determines the veracity of the blood culture results in light of the patient’s condition and analyses the test results to ascertain whether the culture results are contaminated, colonized or infected. The quality control target for this indicator is set at 100%. The timing of blood culture collection and the correct handling of positive results by the department were evaluated by a panel of experts.

### 2.7 Hierarchical management of antimicrobials

According to the characteristics of antibacterial drugs, clinical efficacy, bacterial resistance, adverse reactions, as well as local socio-economic conditions, drug prices and other factors, antibacterial drugs are classified into three categories: non-restricted grade antimicrobials, restrictive grade antimicrobials and special-grade antimicrobials for hierarchical management (Supplementary Table S2). The attending physician and above have the medical advice and prescription authority for restrictive grade antimicrobials, while the deputy chief physician and above have the medical advice and prescription authority for special-grade antimicrobials. The delivery rate equals the delivery number divided by the total number of corresponding antibiotic categories multiplied by 100%.

### 2.8 Intensity of antimicrobial use

The intensity of antimicrobial use is calculated by dividing the cumulative number of defined daily doses (DDDs) of antimicrobial drugs used in within a specific time period by the total number of inpatient days during that period and then multiplying the result by 100%. Inpatient antimicrobial use refers to the sum of DDDs of all antimicrobial used during the same period. The number of inpatient days in the department for the same period is calculated by multiplying the average number of inpatient days in the department by the number of admissions during the same period.

### 2.9 Data collection

Export relevant information on blood culture specimen submissions from the KR-Lis V5.0 hospital information system (Manufacturer: Guangdong Kangruan Technology Co., Ltd.) and the BD BACTEC™ Blood Culture System (Manufacturer: Becton, Dickinson and Company). The data included the following details: case number, department, specimen serial number, machine number, application time, sampling collection time, submission time, acceptance time by the laboratory, report time, and records of any rejected non-conforming specimens. Extract relevant medical records from the electronic medical record system V6.0 (Manufacturer: Beijing Jiahe Meikang Information Technology Co., Ltd.) for review, including the case number and admission date. Export blood culture specimen submission data and relevant information for hospitalized patients prior to antimicrobial therapy from the hospital smart infection management system (Manufacturer: Shanghai Lian Information Technology Co., Ltd.), including: department, number of patients receiving antimicrobial therapy at different levels, number of blood culture submissions prior to therapeutic medication, and the submission rate. Export antimicrobial usage intensity data for hospitalized patients from the Yueke Hospital Information System (Manufacturer: Guangdong Provincial Science and Technology Basic Condition Platform Center), including department, quantity of antimicrobial usage, and the number of patient-days for hospitalized patients during the same period.

### 2.10 Statistical analyses

All statistical analyses were performed with SPSS statistical software, version 29.0 Categorical variables were compared using the chi-square test. For continuous variable that followed a normal distribution, the mean ± standard deviation was used to describe them, and the differences between the groups were assessed using Student’s t test. For continuous variable that did not conform to a normal distribution, the median ± geometric standard deviation or median was used for description. All statistical analyses were evaluated at the statistical significance level of *p* < 0.05 (two-sided).

## 3 Results

### 3.1 Specimen delivery time

**Figure 1:**
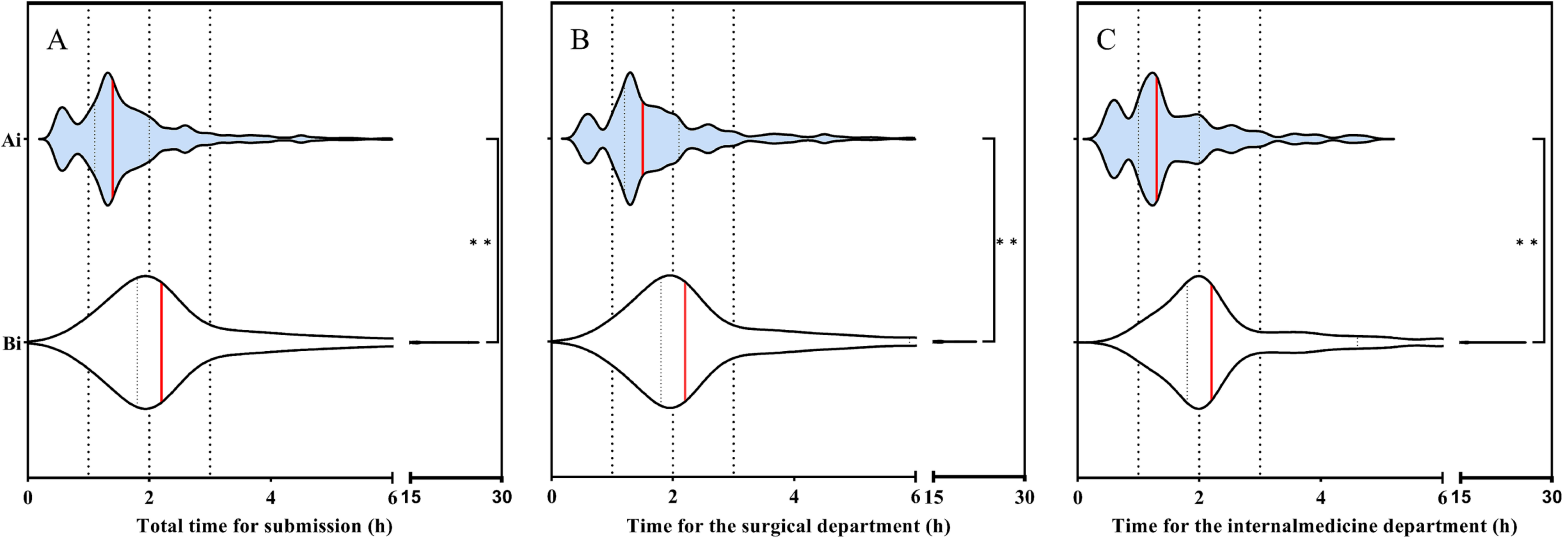
**(A)** Changes in blood culture specimen submission time before and after intervention. **(B)** Changes in blood culture specimen submission time before and after intervention in the surgical department. **(C)** Changes in blood culture specimen submission time before and after intervention in the internal medicine department. Ai: After intervention; Bi: Before intervention. The red line represents the median. Student’s *t* test was used to evaluate the differences between each item. Symbols for *p* values: * < 0.05, ** < 0.001.

The time of submission of blood culture specimens before and after the intervention is shown in Figure 1. The total median submission time before the intervention was 2.2 h, while after the intervention, it decreased to 1.3 h, the difference was statistically significant (*p*< 0.001). Before the intervention, the median time for the delivery of blood culture specimens was 2.2 h in both the internal medicine and surgical departments. After the intervention, this time decreased to 1.3 h in the internal medicine department and 1.5 h in the surgical department, with the difference being statistically significant (*p* < 0.001). This variation is related to the hospital’s layout: the internal medicine and surgical departments are located in separate buildings, with the internal medicine department being closer to the clinical laboratory.

### 3.2 Rates of quality control indicators

The rates of quality control indicators are shown in Table 1. Before the intervention, the blood culture positivity rate was 8.6%, which increased to 9.9% after the intervention (*p* = 0.008). The contamination rate decreased from 1.4% before the intervention to 0.9% after the intervention (*p* = 0.002). Furthermore, the disqualification rate before the intervention was reduced from 1.7 to 1.3% after the intervention (*p* = 0.034). All of these differences were found to be statistically significant. The intervention did not yield a statistically significant difference in the rate of obtaining 2–3 sets of adult blood cultures, which remained above 97% in both before and after intervention groups. After the intervention, the rates of correctly timed blood culture sampling increased from 89.6 to 98.7% (*p* < 0.001). Furthermore, the correct handling of positive results obtained from blood cultures in clinical departments increased from 92.3 to 98.1%, the difference between these two indicators was statistically significant (*p* < 0.001).

**Table 1:** Comparing quality control indicators before and after intervention.

Group	Positivity (%)	Contamination rate (%)	Disqualification rate (%)	Adult blood culture 2–3 sets per episode rate (%)	Rate of correct timing (%)	Correct processing rate (%)
After intervention	9.9 (666/6,720)	0.9 (58/6,720)	1.3 (167/12,435)	97.7 (2,817/2,882)	98.7 (473/479)	98.1 (470/479)
Before intervention	8.6 (557/6,494)	1.4 (93/6,494)	1.7 (203/12,141)	97.7 (2,788/2,853)	89.6 (397/443)	92.3 (409/443)
*p*-value	0.008	0.002	0.034	0.95	<0.001	<0.001

*p-value derived from chi-square test between the before and after intervention group.*

### 3.3 Prevalence of blood culture specimens sent for testing prior to antimicrobial therapy in inpatients

The prevalence of blood culture specimens sent for testing prior to antimicrobial therapy in inpatients is shown in Table 2. When comparing the before and after intervention periods, there was an increase in the delivery rate of blood culture specimens prior to antimicrobial therapy. Specifically, the total delivery rate increased from 15.2% before the intervention to 16.1% after the intervention. Furthermore, the delivery rate for the restrictive-grade increased from 22.9% before the intervention to 26.7% after the intervention. The difference between these two rates was found to be statistically significant (*p* = 0.039 and *p* < 0.001, respectively). There was no statistically significant difference between the rates of blood culture specimens sent before and after intervention with special-grade antimicrobials (*p* = 0.853). However, the proportion of special grade use decreased from 10.9 to 9.0%, which was statistically significant (*p* < 0.001).

**Table 2:** Usage ratios of different levels of antimicrobial agents and pre-treatment blood culture submission status before and after intervention.

	Therapeutic		Restrictive grade		Special grade	
Group	Total number	Delivery rate (%)	Ratio of use (%)	Delivery rate (%)	Ratio of use (%)	Delivery rate (%)
After intervention	12,440	16.1 (2,008/12,440)	55.2 (6,864/12,440)	26.7 (1,831/6,864)	9.0 (1,123/12,440)	57.4 (645/1,123)
Before intervention	11,290	15.2 (1,712/11,290)	58.7 (6,630/11,290)	22.9 (1,515/6,630)	10.9 (1,235/11,290)	57.8 (714/1,235)
*p* - value		0.039	<0.001	<0.001	<0.001	0.853

*p-value derived from chi-square test between the before and after intervention group.*

### 3.4 Intensity of antimicrobial use

When comparing the antimicrobial use before the intervention to the antimicrobial use after the intervention, it was found that antimicrobial use decreased in 31 departments while increasing in 12 departments (Supplementary Table S3). Overall, there was a 10.7% decrease in antimicrobial use throughout the hospital during the period after the intervention.

### 3.5 KAP of medical staff in blood culture collection before- and after intervention

**Figure 2:**
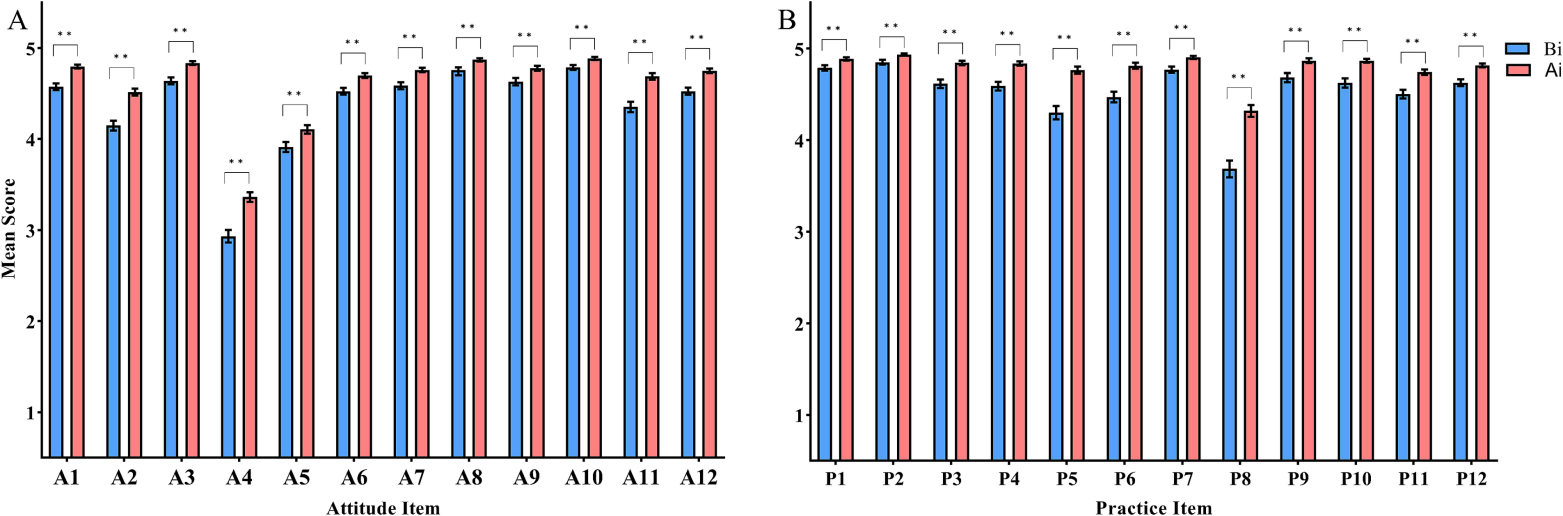
**(A)** Changes in blood culture collection attitude items (A1-A12) among healthcare workers before and after intervention. **(B)** Changes in blood culture collection practice items (P1-P12) among healthcare workers before and after intervention. Ai: After intervention; Bi: Before intervention. Student’s *t* test was used to evaluate the differences between each item. Symbols for *p* values: * < 0.05, ** < 0.001.

The characteristics of the recruited participants are shown in Table 3. There were no significant differences in the gender, age, education level, and title between the before and after intervention groups (*p* > 0.05). Following the intervention, healthcare workers demonstrated a higher level of knowledge on 30 statements compared to the period before intervention (Supplementary Table S4). The percentage of correct answers varied for individual questions. Statement K29, which focused on the handling of blood taken at the catheter, did not show a statistical difference between the before and after intervention group (Supplementary Table S4). The attitude and practice sections comprised 12 attitude and 12 practice items, respectively (Supplementary Table S5). These were presented as statements, and participants were asked to rate them using a Likert scale from 1 (strongly disagree) to 5 (strongly agree). The lowest score in the attitude items was A4, which pertained to the belief that taking blood cultures causes unnecessary pain to patients (Figure 2). The lowest score in the practice items was P4, which focused on the environmental requirements for preserving blood cultures when specimens cannot be tested immediately (Figure 2).

**Table 3:** The characteristics of healthcare workers.

Characteristics	Before intervention (*n* = 1,031)	After intervention (*n* = 1,558)	*p*-value
Gender, *n* (%)			0.07
Male	211 (20.47)	366 (23.49)	
Female	820 (79.53)	1,192 (76.51)	
Age (years), *n* (%)			0.25
20–30	471 (45.68)	695 (44.61)	
31–40	370 (35.89)	538 (34.53)	
41–50	151 (14.65)	241 (15.47)	
>50	39 (3.78)	84 (5.39)	
Educational level, *n* (%)			0.13
Junior/ high school and below	192 (18.63)	275 (17.65)	
College degree	631 (61.20)	907 (58.22)	
Postgraduate and above	208 (20.17)	376 (24.13)	
Title, *n* (%)			0.63
Primary	581 (56.35)	880 (56.48)	
Intermediate	256 (24.83)	366 (23.49)	
Senior	194 (18.82)	312 (20.03)	

*p-value derived from chi-square test between the before and after intervention group.*

## 4 Discussion

With the successful implementation of a multidisciplinary intervention in a tertiary referral hospital in Guangdong province, China, we were able to reduce the submission time significantly for blood culture specimens from 2.2 h to 1.3 h. This reduction in time aligns with the recommendation that blood culture specimens should be transported to the laboratory within 2 h once collected (16, 17). However, some clinical departments still lack the necessary resources to accomplish this. We found that the delivery time of specimens is longer at night than the daytime, due to the shortage of manpower at night. Therefore, optimization in scheduling testing staff and management, as well as improving the delivery system, was necessary. The information technology department is preparing to develop an app, which like the Uber Eats or Grubhub app. Healthcare workers will place orders, and the software will automatically dispatch the orders to transport workers on duty. Depending on the type of specimen, different priority levels will be assigned for shipping. If a specimen does not arrive on time, the app will automatically alert and warn the transport workers. This could be an effective method to reduce specimen delivery times.

According to the Clinical and Laboratory Standards Institute (CLSI), contamination rate should not exceed an acceptable range, typically ≤3.0% (18). Before intervention, blood culture contamination rate was less than 3%, it was further reduced to 0.86% after the intervention. Therefore, it is possible to achieve and sustain reduce the blood culture contamination rate benchmark of ≤3% in tertiary referral hospitals (19). Drawing multiple sets of blood culture is another important factor in increasing the positivity rate and differentiating between contamination and true bacteremia (4). In several studies, rates of recovery increased with the number of blood culture sets obtained, ranging from 73% with 1 blood culture set to over 99% when 3 sets were obtained (3). By limiting prescriptions to 2–3 sets per episode in the medical prescription system, we can achieve a higher rate of 2–3 sets of blood cultures in adults. Videos of blood culture collection practices, which clinical departments are only required to submit once, highlighted several issues, including: the use of butterfly needle collection with anaerobic vials drawn first followed by aerobic vials; operations performed without sterile gloves; lack of skin cleansing and incorrect disinfection steps; failure to use alcohol for deiodination; lack of shaking after blood draws; and failure to implement effective procedures.

Research has shown that the implementation of evidence-based clinical guidance, along with provider education and feedback regarding blood culture best practices in the medical ICU (MICU) and medicine units at a large academic center, reduced blood culture utilization by 18 and 30%, respectively, while the proportion of solitary blood culture remained similar (MICU) or decreased (medicine units) (20). So, continuous education and feedback to the healthcare workers were good methods to reduce these problems. Providing basic education on elements for proper blood drawing technique and improving worker skill, competency training, and procedure were reasons for good outcome.

Obtaining blood cultures prior to administering antimicrobials is considered best practice according to international guidelines for patients with suspected infections (21). Awareness of the importance of blood cultures has increased among healthcare professionals, who are now proactively pursuing accurate blood culture results to make targeted infection control decisions. Providing timely and accurate blood cultures results to the clinic enables physicians to promptly adjust or optimize antimicrobial regimens, reduce the use of non-specific broad-spectrum antimicrobials, lower the risk of bacterial resistance, and improve treatment outcomes. Otherwise, antimicrobial therapy may reduce blood culture positivity. Therefore, if cultures are not obtained before antimicrobial administration, they should be collected as soon as possible afterward (22).

Through the questionnaire survey, it was found that some healthcare workers mistakenly feel that blood culture specimens were held be refrigerated or frozen. This wrong operation may kill some of the microorganisms. Therefore, it is necessary to optimize the process of handling blood culture specimens and improve training in this knowledge. One potential strategy to address this issue could be to place educational fliers or posters on refrigerator doors.

Antimicrobial resistance would have been the 12th leading the Global Burden of Diseases Level 3 cause of death globally, ahead of both HIV and malaria. It is essential to minimize the use of antibiotics when they are not necessary to improve human health, such as treating viral infections, should be prioritized. To this end, building infrastructure that allows clinicians to diagnose infection accurately and rapidly is crucial so that antimicrobial use can be narrowed or stopped when appropriate (23, 24). Multidisciplinary interventions aimed at improving blood culture efficiency can enhance the positivity rate, reduce contamination rates, and provide better evidence for clinicians to make informed treatment decisions, thereby optimizing antimicrobial use and sustainably reducing antibiotic resistance.

There are several limitations in this study. The study does not include data on patient outcomes such as mortality rates, length of hospital stay, and readmission rates, which would provide a more comprehensive evaluation of the interventions’ impact. Additionally, the follow-up period may be too short to adequately assess the long-term effects of the interventions on antimicrobial resistance patterns. These factors should be considered when interpreting the findings and evaluating the overall impact of the interventions.

## 5 Conclusion

Multidisciplinary interventions can improve blood culture efficiency and optimize antimicrobial utilization.

## Data Availability

The original contributions presented in the study are included in the article/Supplementary material, further inquiries can be directed to the corresponding author.
