# Supplementary material for: Effectiveness of multidisciplinary interventions to improve blood culture efficiency and optimize antimicrobial utilization
Source: Front Public Health. 2024 Oct 4;12:1432433. doi: 10.3389/fpubh.2024.1432433 (PMC11486708; doi:10.3389/fpubh.2024.1432433)
Supplement: Supplementary file 1 [file Table_1.DOC]

Supplementary Material

**Effectiveness of multidisciplinary interventions to improve blood culture efficiency and optimize antimicrobial utilization**

Zihuan Li^1^, Keqi Hu^2^, Tian Wang^1^, Baohong Liu^1^, Wen Zheng^3^, Jianqun Zhou^4^, Ting Fan^1^, Maorui Lin^5^, Guanwen Lin^1^, Sujuan Li^6^, Cuiqiong Fan^1*^

^1^Department of Infection Prevention and Control, Guangdong Second Provincial General Hospital, Guangzhou, China

^2^Department of Science and Education, Guangdong Second Provincial General Hospital, Guangzhou, China

^3^Department of Nursing, Guangdong Second Provincial General Hospital, Guangzhou, China

^4^Department of Thyroid and Breast Surgery, Guangdong Second Provincial General Hospital, Guangzhou, China

^5^Department of Laboratory Medicine, Guangdong Second Provincial General Hospital, China

^6^Department of Pharmacy, Guangdong Second Provincial General Hospital, Guangzhou, China

*** Correspondence:**Cuiqiong Fan
497950956@qq.com

Number of pages: 15

Number of tables: 5

Number of figures: 1

**Table of Contents**

Table S1 S3-S4

Table S2 S5-S6

Table S3 S7-S8

Table S4 S9-S13

Table S5 S14-S15

Figure S1 S16

Table S1: Multidisciplinary collaboration model: How did it all come together?

| **Multidisciplinary Team** | **How did it all come together** |
| --- | --- |
| **Medical Departments** |  |
|  | 1.Under the leadership of the Department of Infection Prevention and Control, establish a multi-departmental cooperation model to clarify the responsibilities of each department. |
|  | 2.Hold a monthly joint meeting. |
|  | 3.Regularly conduct departmental supervision inspections to verify the implementation of corrective actions. |
|  | 4.Regularly release microbiological surveillance data, issue reminders and public notices for departments that do not meet standards, and offer suggestions for improvement. |
|  | 5.Offer training and assessment on the submission of microbiological specimens and the appropriate utilization of antibiotics. |
|  | 6.Establish and maintain the hospital's antimicrobial stewardship formulary. |
|  | 7.Conduct multidisciplinary consultations focused on managing complex cases of infectious diseases. |
| **Nursing Department** |  |
|  | 1.Conduct on-site inspections of specimen submission processes, collection methods, site identification, collection indications, and specimen volumes. |
|  | 2.Organize the production of a video demonstrating the standardized collection of blood culture samples for adults or children in each department. |
|  | 3.Establish a blood culture specimen collection nursing standard management team, which includes liaison officers from the hospital infection team of each ward. |
| **Laboratory Department** |  |
|  | 1.Regularly provides feedback on microbiological assay performance metrics. |
|  | 2.Developing standardized processes for specimen collection, including indications, methods, storage, and transport procedures. |
|  | 3.Establishes a critical value reporting system to promptly notify clinical departments of positive blood culture results. |
| **Pharmacy Department** |  |
|  | 1.Conducts regular reviews of antimicrobial prescriptions and oversees the appropriateness of clinical antimicrobial use. |
| **Information Department** |  |
|  | 1.Establishes interoperability between the case system, nursing system, and laboratory system to enable real-time monitoring, alerting, collection, analysis, and reporting functions for microbiological specimen submission. |
| **Distribution Centre** |  |
|  | 1.Strengthens training efforts to enhance responsibility and implements a system of rewards and penalties. |

Table S2: The Category of Antimicrobial Drug Classification.

| Categories | Non-restricted grade | Restrictive grade | Special-grade |
| --- | --- | --- | --- |
| **Penicillin** |  |  |  |
|  | Amoxicillin | Piperaclilin Sodium and Tazobactam Sodium |  |
|  | Amoxicillin and Clavulanate Potassium |  |  |
| **Cephalosporins** |  |  |  |
|  | Cefalexin | Ceftazidime | Ceftazidime/avibactam |
|  | Cephazolin | Cefixime | Cefepime |
|  | cefaclor | Cefdinir |  |
|  | Cefuroxime | Ceftriaxone |  |
|  |  | Sulbactam and Cefoperazone |  |
| **Other Beta-lactam antibiotic** |  |  |  |
|  |  | Cefoxitin | Imipenem/cilastatin |
|  |  | Aztreonam | Meropenem |
|  |  | Ertapenem |  |
| **Aminoglycosides** |  |  |  |
|  | Gentamicin |  |  |
|  | Amikacin |  |  |
| **Tetracyclines** |  |  |  |
|  |  | Minocycline | Tigecycline |
|  |  | Doxycycline |  |
| **Macrolides** |  |  |  |
|  | Clarithromycin | Azithromycin |  |
|  | Roxithromycin |  |  |
| **Other Antibiotics** |  |  |  |
|  | Clindamycin | Chloramphenicol | Vancomycin |
|  |  |  | Teicoplanin |
|  |  |  | Linezolid |
| **Quinolones** |  |  |  |
|  | Norfloxacin | Moxifloxacin |  |
|  | Levofloxacin | Sitafloxacin |  |
|  | Ciprofloxacin |  |  |
| **Nitroimidazoles** |  |  |  |
|  | Metronidazole |  |  |
|  | Tinidazole |  |  |
| **Other Antibiotics** |  |  |  |
|  |  | Fosfomycin | Polymyxin B |
|  |  |  | Colistimethate Sodium |
| **Antifungal drugs** |  |  |  |
|  | Nystatin | Fluconazole | Amphotericin B |
|  | Itraconazole | Posaconazole | Voriconazole |
|  |  | Isavuconazole | Caspofungin |

Table S3: The intensity of antimicrobial use, measured in defined daily doses (DDDs) per 100 patient-days, was assessed before and after intervention in whole hospital departments.

| No. | Department | Before intervention | | |  | After intervention | | | Ratio |
| --- | --- | --- | --- | --- | --- | --- | --- | --- | --- |
|  |  | DDDs | Inpatient days | Intensity |  | DDDs | Inpatient days | Intensity |  |
| 1 | Maternity ward | 743.12 | 5284 | 14.06 |  | 1423.81 | 7001 | 20.34 | 44.61% |
| 2 | Traumatology and orthopedics | 3790.3 | 9534 | 39.76 |  | 3088.84 | 11443 | 26.99 | -32.10% |
| 3 | Pediatrician | 1800.57 | 8708 | 20.68 |  | 3917.75 | 11992 | 32.67 | 58.00% |
| 4 | Otorhinolaryngology, Head and Neck Surgery | 1825.74 | 5582 | 32.71 |  | 2802.26 | 7065 | 39.66 | 21.27% |
| 5 | Rheumatology and Immunology | 1973.04 | 7807 | 25.27 |  | 1797.47 | 7254 | 24.78 | -1.95% |
| 6 | Otolaryngology | 1930.35 | 5128 | 37.64 |  | 2590.29 | 6424 | 40.32 | 7.12% |
| 7 | Hepatobiliary and Pancreatic Hernia Surgery | 3091.76 | 7165 | 43.15 |  | 3732.57 | 8285 | 45.05 | 4.41% |
| 8 | Infection (Hepatology) | 1476.21 | 2481 | 59.5 |  | 1465.27 | 2695 | 54.37 | -8.62% |
| 9 | Joint orthopedics | 2215.13 | 7396 | 29.95 |  | 1398.78 | 7013 | 19.95 | -33.40% |
| 10 | Respiratory and Critical Care Medicine | 17979.89 | 10887 | 165.15 |  | 18674.71 | 11219 | 166.46 | 0.79% |
| 11 | Emergency | 395.46 | 1140 | 34.69 |  | 427.76 | 1391 | 30.75 | -11.35% |
| 12 | Orthopedic spine | 1450.23 | 6027 | 24.06 |  | 1686.77 | 6643 | 25.39 | 5.53% |
| 13 | Thyroid and Breast Surgery | 101.14 | 2684 | 3.77 |  | 83.84 | 3132 | 2.68 | -28.96% |
| 14 | Interventional vascular medicine | 238.22 | 2365 | 10.07 |  | 192.65 | 2353 | 8.19 | -18.72% |
| 15 | Rehabilitation Medicine | 87.23 | 4182 | 2.09 |  | 89.81 | 5314 | 1.69 | -18.97% |
| 16 | Dentistry | 27.67 | 93 | 29.75 |  | 18.67 | 51 | 36.6 | 23.03% |
| 17 | Urology | 5492.19 | 8788 | 62.5 |  | 5980.58 | 9831 | 60.83 | -2.66% |
| 18 | Endocrinology | 1625.65 | 7603 | 21.38 |  | 787.38 | 7016 | 11.22 | -47.51% |
| 19 | Dermatology | 74.75 | 296 | 25.25 |  | 43.85 | 257 | 17.06 | -32.43% |
| 20 | Organ Transplantation Unit | 1042.36 | 2115 | 49.28 |  | 843.21 | 2000 | 42.16 | -14.45% |
| 21 | Family Medicine | 1197.93 | 3565 | 33.6 |  | 1107.29 | 3912 | 28.31 | -15.77% |
| 22 | Neurology | 5226.77 | 17531 | 29.81 |  | 3041.96 | 17660 | 17.23 | -42.23% |
| 23 | Neurosurgery | 3566.41 | 9690 | 36.81 |  | 3224.52 | 9405 | 34.29 | -6.85% |
| 24 | Nephrology | 1562.42 | 5716 | 27.33 |  | 1508.76 | 10102 | 14.94 | -45.36% |
| 25 | Centre for Reproductive Medicine | 55.17 | 129 | 42.76 |  | 22 | 16 | 137.5 | 221.53% |
| 26 | Pain medicine | 203.31 | 2008 | 10.12 |  | 174.44 | 2227 | 7.83 | -22.64% |
| 27 | Gastrointestinal Surgery | 3359.9 | 8648 | 38.85 |  | 4065.36 | 8910 | 45.63 | 17.44% |
| 28 | Microprosthetic Orthopedics | 951.11 | 1990 | 47.79 |  | 2648.75 | 6254 | 42.35 | -11.39% |
| 29 | Gastroenterology | 1201.43 | 6804 | 17.66 |  | 1529.74 | 8707 | 17.57 | -0.50% |
| 30 | Psycho-psychiatry | 97.23 | 4504 | 2.16 |  | 87.45 | 4475 | 1.95 | -9.47% |
| 31 | Cardiothoracic surgery | 3919.33 | 7137 | 54.92 |  | 2990.51 | 7175 | 41.68 | -24.10% |
| 32 | Cardiovascular Medicine II | 1118.22 | 8174 | 13.68 |  | 626.13 | 7284 | 8.6 | -37.16% |
| 33 | Cardiovascular Medicine III | 825.92 | 3566 | 23.16 |  | 541.1 | 3306 | 16.37 | -29.33% |
| 34 | Cardiovascular Medicine I | 1696.33 | 8475 | 20.02 |  | 969.85 | 7121 | 13.62 | -31.96% |
| 35 | Cardiovascular Surgery | 1007.19 | 1772 | 56.84 |  | 724.3 | 1955 | 37.05 | -34.82% |
| 36 | Cardiology | 3754.23 | 5043 | 74.44 |  | 2930.94 | 4538 | 64.59 | -13.24% |
| 37 | Hematology | 7181.21 | 6664 | 107.76 |  | 5952.54 | 6581 | 90.45 | -16.06% |
| 38 | Ophthalmology | 28.88 | 981 | 2.94 |  | 32.43 | 1153 | 2.81 | -4.43% |
| 39 | Plastic surgery | 134.31 | 806 | 16.66 |  | 132.98 | 1110 | 11.98 | -28.11% |
| 40 | Chinese medicine | 205.5 | 2697 | 7.62 |  | 54.96 | 3134 | 1.75 | -76.99% |
| 41 | Oncology II | 1000.25 | 5808 | 17.22 |  | 1135.24 | 7145 | 15.89 | -7.74% |
| 42 | Oncology I | 1016.81 | 7503 | 13.55 |  | 819.73 | 5967 | 13.74 | 1.37% |
| 43 | Critical care medicine | 5740.62 | 3071 | 186.93 |  | 4908.71 | 2298 | 213.61 | 14.27% |
| 44 | **Total** | 92411.46 | 227547 | 40.61 |  | 90275.98 | 248814 | 36.28 | -10.66% |

Table S4: Analysis of healthcare workers' knowledge awareness on blood culture collection before and after intervention.

| **Knowledge statements.** | **Before intervention [n (%)]** | | |  | **After intervention [n (%)]** | | | ***p*-value** |
| --- | --- | --- | --- | --- | --- | --- | --- | --- |
|  | **Doctor (n=335)** | **Nurse (n=696)** | **Overall (1031)** |  | **Doctor (n=562)** | **Nurse (n=996)** | **Overall (1558)** |  |
| K1: A blood culture is what is sent from a patient to a microbiology laboratory for culture? (Blood or sterile body fluids) | 104（31.0） | 183（26.3） | 287（27.8） |  | 314（55.9） | 570（57.2） | 884（56.7） | < 0.05 |
| K2: How do pathogenic microorganisms and their products exist in the circulating blood during bloodstream infections, causing damage to the organism and, in severe cases, leading to shock or even death? (transient，indirect，sustainable) | 173（51.6） | 440（63.2） | 613（59.5） |  | 534（95.0） | 953（95.7） | 1487（95.4） | < 0.05 |
| K3: Who takes blood culture specimens from patients? (Nurse) | 212（63.3） | 441（63.4） | 653（63.3） |  | 525（93.4） | 942（94.6） | 1467（94.2） | < 0.05 |
| K4: Which groups of people can be prescribed a medical order for blood culture collection? (Adults, children and infants) | 330（98.5） | 665（95.6） | 995（96.5） |  | 558（99.3） | 991（99.5） | 1549（99.4） | < 0.05 |
| K5: What is the typical volume of blood collected per vial for adult blood cultures? (8-10 ml) | 223（66.6） | 526（75.6） | 749（72.7） |  | 551（98.0） | 986（99.0） | 1537（98.7） | < 0.05 |
| K6: What is the order in which syringe-collected blood is injected into culture bottles? (When there is enough blood volume, anaerobic vials should be injected first, followed by aerobic vials.) | 203（60.6） | 405（58.2） | 608（59.0） |  | 534（95.0） | 953（95.7） | 1487（95.4） | < 0.05 |
| K7: What is the order in which the butterfly-collected blood is injected into the culture flask? (When there is enough blood volume, inject the aerobic vials first, followed by the anaerobic vials.) | 195（58.2） | 472（67.8） | 667（64.7） |  | 538（95.7） | 968（97.2） | 1506（96.66） | < 0.05 |
| K8: What are the precautions for syringe collection? (No need to change needles when injecting syringes into culture flasks) | 162（48.4） | 422（60.6） | 584（56.6） |  | 532（94.7） | 967（97.1） | 1499（96.2） | < 0.05 |
| K9: When is the correct time to collect a blood culture specimen? (Chills, start of fever or before antimicrobial treatment) | 269（80.3） | 450（64.7） | 719（69.7） |  | 544（96.8） | 967（97.1） | 1511（98.0） | < 0.05 |
| K10: What is incorrect about the timing and number of blood cultures collected from patients with acute endocarditis and those with subacute endocarditis? (Blood collection prior to administration) | 163（48.7） | 193（27.7） | 356（34.5） |  | 530（94.3） | 952（95.6） | 1482（95.1） | < 0.05 |
| K11: What is the method of sterilising the tops of culture bottles? (Use alcohol, not iodine) | 186（55.5） | 330（47.4） | 516（50.1） |  | 421（74.9） | 740（74.3） | 1161（74.5） | < 0.05 |
| K12: What is an adult one copy/set of blood cultures? ( 1 aerobic flask + 1 anaerobic flask) | 68（20.3） | 191（27.4） | 259（25.1） |  | 481（85.6） | 880（88.4） | 1361（87.4） | < 0.05 |
| K13: What is the effect of collection method and number of collections on the positive detection rate? (The rate of positive detection of pathogenic bacteria increases with the number of sets collected.) | 86（25.7） | 255（36.6） | 341（33.1） |  | 528（95.0） | 943（94.7） | 1471（94.4） | < 0.05 |
| K14: What is the correct statement about the timing of blood culture bottle delivery? (The specimen should be sent to the laboratory as soon as possible after collection, preferably within two hours.) | 273（81.5） | 573（82.3） | 846（82.1） |  | 537（95.6） | 964（96.8） | 1501（96.3） | < 0.05 |
| K15: What is the proper way to handle culture bottles when they are not delivered to the laboratory in a timely manner? (Keep at room temperature) | 73（21.8） | 199（28.6） | 272（26.4） |  | 406（72.2） | 779（78.2） | 1185（76.1） | < 0.05 |
| K16: What is the correct statement about the time and site of specimen collection? (Ideally, it is recommended to collect two or more specimens from different anatomical sites simultaneously.) | 230（68.7） | 365（52.4） | 595（57.7） |  | 546（97.2） | 957（96.1） | 1503（96.5） | < 0.05 |
| K17: What is the correct statement regarding skin disinfection at the puncture site prior to blood culture collection? (Disinfect in a circular motion starting from the puncture point and moving outwards. Ensure that the area disinfected is at least 3cm in diameter.) | 97（30.0） | 97（13.9） | 194（18.8） |  | 475（84.5） | 802（80.5） | 1277（83.0） | < 0.05 |
| K18: What is a correct statement for a physician to make when prescribing a blood culture? (Blood cultures are prescribed according to the needs of different diseases.) | 216（64.5） | 435（62.5） | 651（63.1） |  | 524（93.2） | 945（94.9） | 1469（94.3） | < 0.05 |
| K19: What is the clinical significance of blood cultures? (Clarifying the Basis for Treatment and so on) | 254（75.8） | 549（78.9） | 803（77.9） |  | 516（91.8） | 957（96.1） | 1473（94.5） | < 0.05 |
| K20: What is the significance of a positive blood culture? (Optimization of antimicrobials and so on ) | 289（86.3） | 527（75.7） | 816（79.2） |  | 533（94.8） | 930（93.4） | 1463（93.9） | < 0.05 |
| K21: What are the considerations for collecting blood cultures from patients with suspected infections? (multi-organ failure, elevated inflammatory response parameters and so on) | 249（74.3） | 375（53.9） | 624（60.5） |  | 549（97.7） | 965（96.9） | 1514（97.2） | < 0.05 |
| K22: What are the precautions for blood culture collection? (Information to be indicated on the label) | 47（14.0） | 172（24.7） | 219（21.2） |  | 528（95.0） | 931（93.5） | 1459（93.7） | < 0.05 |
| K23: What is the correct statement about the amount of blood collected for blood cultures in infants and children? (The amount of blood collected from infants and children should not exceed 1% of their total blood volume. Please refer to the instructions.) | 106（31.6） | 280（40.2） | 386（37.4） |  | 414（73.7） | 727（74.0） | 1141（73.2） | < 0.05 |
| K24: What is the correct statement for the collection of catheter-related bloodstream infections? (Blood was collected simultaneously from the indwelling catheter and peripheral vein without removing it, and one set of each was sent for blood culture.) | 132（39.4） | 405（58.2） | 537（52.1） |  | 337（60.0） | 693（69.6） | 1030（66.1） | < 0.05 |
| K25: What is a correct statement about blood culture administration? (Standard operating procedures can effectively reduce contamination rates) | 166（49.6） | 242（34.8） | 408（39.6） |  | 496（88.3） | 884（88.8） | 1380（88.6） | < 0.05 |
| K26: What is the correct method of blood culture collection? (Recommended for venous blood collection) | 112（33.4） | 282（40.5） | 394（38.2） |  | 468（83.3） | 822（82.5） | 1290（82.8） | < 0.05 |
| K27: Should blood be collected from patients with infective endocarditis every 8 hours, with intervals of at least half an hour? (True) | 308（91.9） | 545（78.3） | 853（82.7） |  | 557（99.1） | 991（99.5） | 1548（99.4） | < 0.05 |
| K28: Do you think the difference between leaving blood cultures for children and adults is mainly the difference in the amount of blood taken, but nothing else? (False) | 188（56.1） | 307（44.1） | 495（48.0） |  | 510（90.8） | 910（91.4） | 1420（91.1） | < 0.05 |
| K29: Do you think the fluid inside the catheter needs to be discarded if blood needs to be taken from the catheter? (False) | 140（41.8） | 234（33.6） | 374（36.3） |  | 248（44.1） | 342（34.3） | 590（37.9） | 0.41 |
| K30: If a patient with iodine allergy is encountered during the operation, it is possible to sterilize the area twice with alcohol and allow it to dry before puncturing for blood collection. (True) | 314（93.7） | 644（92.5） | 958（92.9） |  | 552（98.2） | 988（99.2） | 1540（98.9） | < 0.05 |

Table S5: The questions of attitude and practice sections.

| **Section** | **Question** |
| --- | --- |
| **Attitude** |  |
|  | A1: I feel that blood culture collection is an important tool in the diagnosis of infectious diseases. |
|  | A2: I feel blood culture collection is necessary, even if the patient already has other diagnostic results. |
|  | A3: Before taking blood cultures, it is important to communicate fully with the patient and obtain their consent. |
|  | A4: I feel that taking blood cultures causes unnecessary pain to patients. |
|  | A5: I feel taking blood cultures is a very low risk for the patient. |
|  | A6: I feel blood cultures should be taken as soon as necessary. |
|  | A7: I feel that collecting blood samples correctly minimizes complications in blood culture collection. |
|  | A8: I feel that strict adherence to standardized and regulated procedures can maximize the rate of positive blood culture detection. |
|  | A9: I feel that staff must follow strict principles of sterilization and asepsis when performing blood culture collections. |
|  | A10: I feel that sterile gloves should be worn when performing blood culture collection. |
|  | A11: I am willing to receive training in relevant techniques for collecting blood cultures and related knowledge. |
|  | A12: I feel that healthcare professionals should adhere to relevant norms and standards when performing blood culture collection. |
| **Practice** |  |
|  | P1: I am capable of consciously adhering to the relevant norms and standards when collecting blood cultures. |
|  | P2: Before using disposable medical devices, it is important to check that the packaging is intact, uncontaminated, and within the expiry date. |
|  | P3: Sterile gloves will be worn during blood culture collection, and strict hand hygiene will be practiced. |
|  | P4: In my blood culture collections, the bottle tops are sterilized using 75% alcohol and left to dry for 60 seconds before the specimen is taken. |
|  | P5: Blood culture specimens are drawn using a syringe and injected first into an anaerobic vial and then into an aerobic vial once there is a sufficient amount of blood. |
|  | P6: Butterfly blood collection is used to draw blood culture specimens. Aerobic vials are filled first, followed by anaerobic vials when there is sufficient blood volume. |
|  | P7: A blood culture specimen should be sent for testing as soon as possible after collection, within 30 minutes and no later than 2 hours. |
|  | P8: If the blood culture specimen cannot be sent promptly after collection, it will be stored at room temperature until submission. |
|  | P9: Blood cultures were collected after disinfecting the skin puncture site with an antiseptic solution, covering an area of at least 3cm in diameter. |
|  | P10: The culture bottle was gently shaken after blood collection to prevent blood clotting. |
|  | P11: I would like to attend needle stick injury prevention training. |
|  | P12: I am capable of performing the necessary blood culture collection practices as per my training. |

Figure S1: Blood Culture Specimen Collection Process.


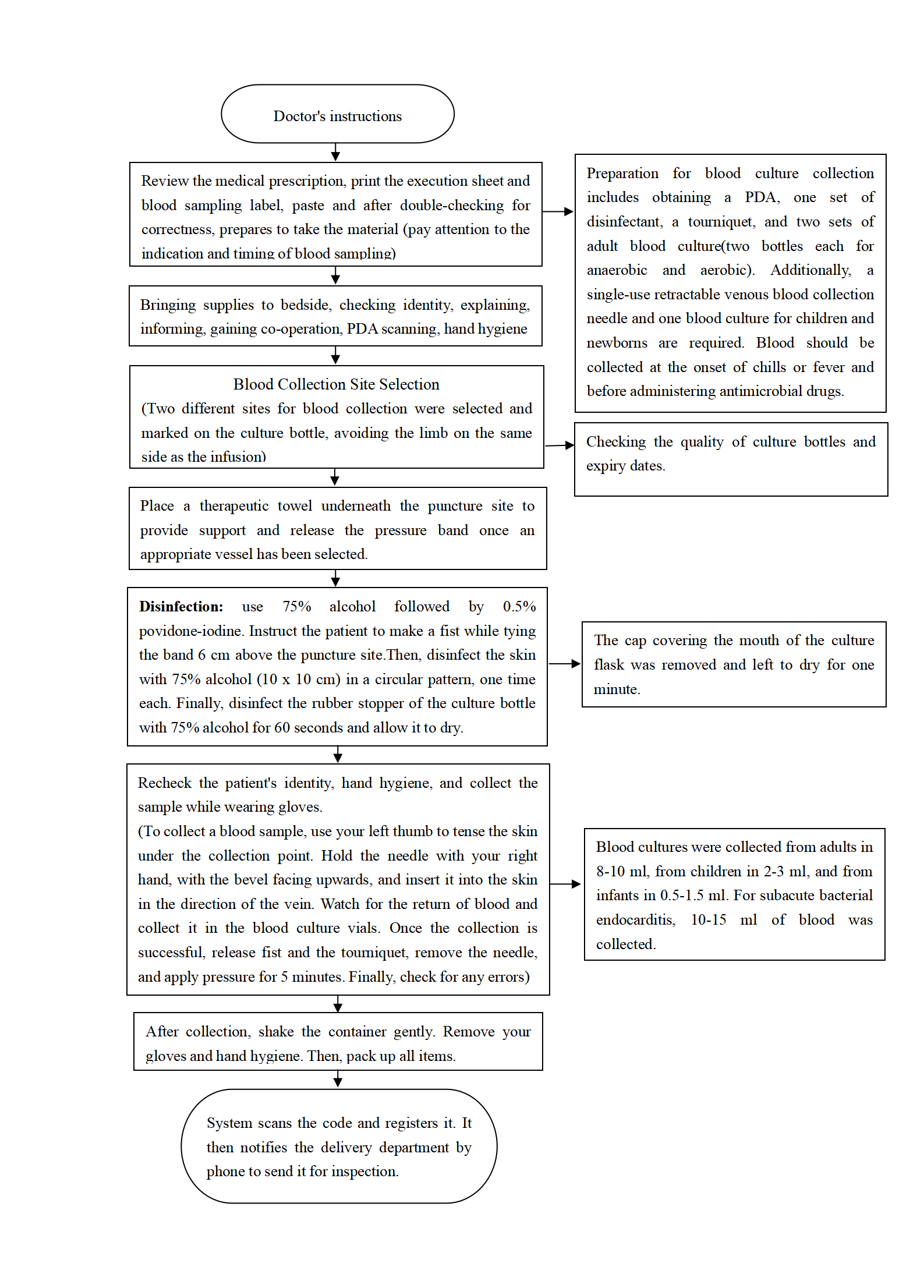


**Footnote:** PDA (Personal Digital Assistant) is a mobile device specifically designed for the medical field, featuring powerful data collection, processing, and transmission capabilities.
